# Herpes zoster and meningitis in an immunocompetent child: a case report

**DOI:** 10.1186/s13256-019-2082-z

**Published:** 2019-06-15

**Authors:** Ryu Yasuda, Kisei Minami, Akira Ogawa, Hideshi Okada, Runa Terakawa, Yumi Koike, Shinji Ogura, Kouichi Takeuchi, Tsukasa Higuchi

**Affiliations:** 1 Department of General Pediatrics, Nagano Children’s Hospital, Nagano, Japan; 2 Department of Emergency and Disaster Medicine, Gifu University Graduate School of Medicine, 1-1 Yanagido, Gifu, 501-1194 Japan

**Keywords:** Herpes zoster, Meningitis, Immunocompetent child, Acyclovir, Varicella zoster virus

## Abstract

**Background:**

Development of neurological complications of varicella zoster virus reactivation is relatively uncommon, particularly in an immunocompetent child.

**Case presentation:**

An 11-year-old Asian girl presented with headache and skin rash on her left chest. She was diagnosed with meningitis, and herpes zoster was confirmed by polymerase chain reaction using cerebrospinal fluid. Acyclovir was administered intravenously. Given the favorable evolution of the clinical course, she was discharged from the hospital on day 8 of her illness. She had no apparent sequelae or comorbidities at the time of the 6-week follow-up.

**Conclusions:**

Neurological complications such as meningitis due to varicella zoster virus reactivation are uncommon, especially in an immunocompetent child; no specific immune deficiency was identified in our patient. We conclude that, although rare, varicella zoster virus should be recognized as a potential cause of viral meningitis in immunocompetent children.

## Background

Varicella zoster virus (VZV) is a member of the herpesvirus family and causes varicella (chickenpox). After primary infection, VZV establishes latency in the cranial nerve and dorsal root ganglia. Cell-mediated immunity to VZV declines in elderly people or in the immunosuppressive state, and it leads to virus reactivation that can cause herpes zoster. Development of neurological complications due to VZV reactivation is considered to be relatively uncommon, particularly in an immunocompetent child. In this report, we describe herpes zoster and meningitis in an immunocompetent girl.

## Case presentation

An 11-year-old Asian girl presented with headache and skin rash on the left side of her chest that had begun 3 days earlier. She had been diagnosed with varicella when she was 2 years old and therefore had no history of receiving the VZV vaccine. She did not have any episode associated with primary immunodeficiency.

Before the onset of illness, she had been feeling fatigue due to exhaustive preparation for a school gymnastics event over the course of several weeks. She visited the local clinic due to repeated afebrile vomiting and severe headache. On the same day, she was admitted to our hospital with a concern of meningitis.

**Fig. 1:**
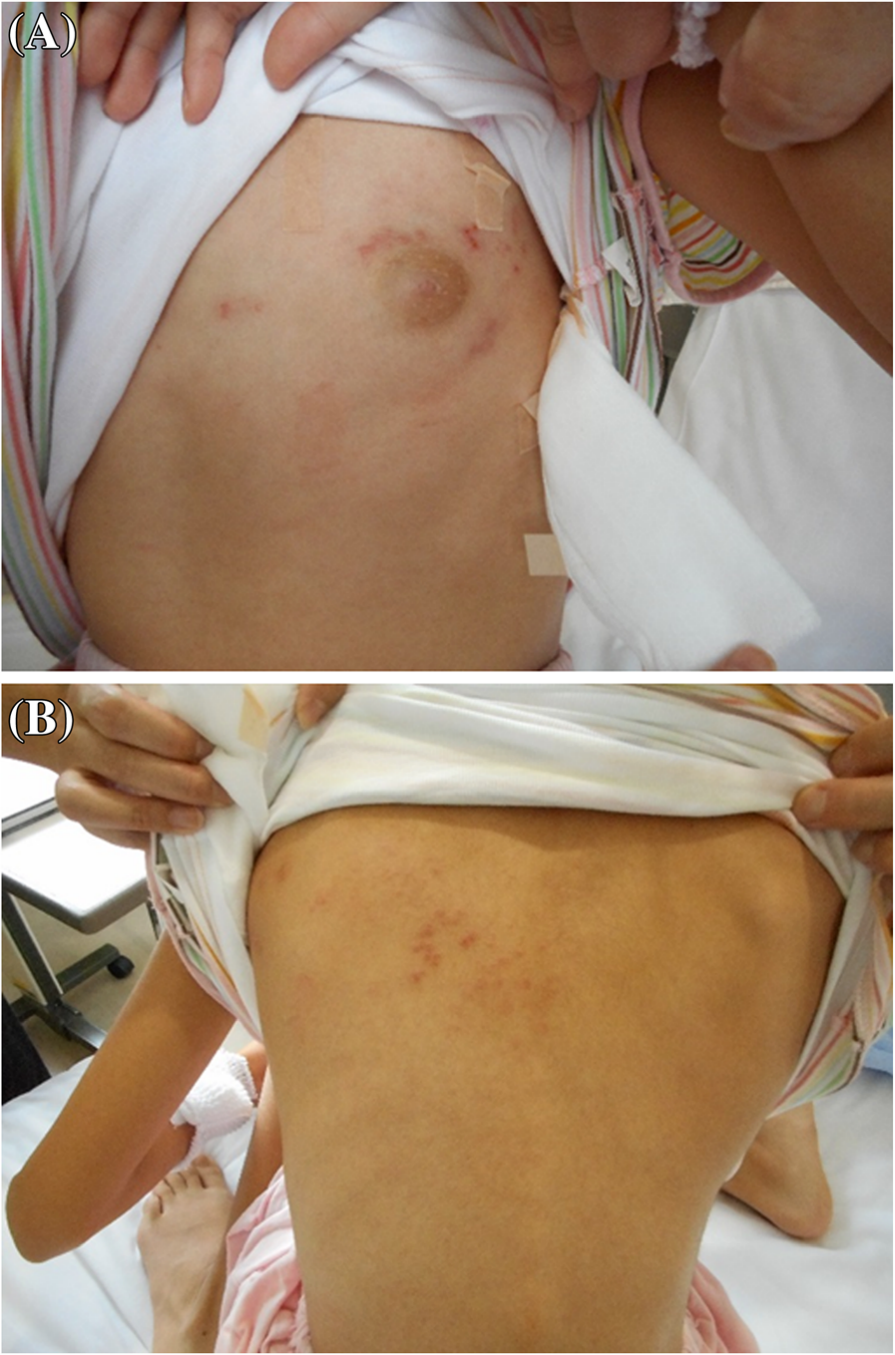
Pictures of maculopapular rash evolving into vesicles with erythematous regions. **a** Ventral side. **b** Dorsal side

Upon her admission, physical examination revealed a body temperature of 37.2 °C, respiratory rate of 20 breaths/min, heart rate of 85 beats/min, and normal hemodynamic parameters with blood pressure of 117/68 mmHg. She was noted to have a maculopapular rash evolving into vesicles with erythematous regions on the left side of her chest (Fig. 1). Her consciousness was clear, and her deep tendon reflexes were normal; Kernig’s sign was negative, although she had neck stiffness.

A cerebrospinal fluid (CSF) examination revealed normal protein concentration (36 mg/dl), normal glucose level (47 mg/dl; blood glucose level, 92 mg/dl), and lymphocytic pleocytosis (429 lymphocytes/μl). Bacterial culture of CSF yielded no growth. Varicella zoster virus (VZV) deoxyribonucleic acid (DNA) was detected in CSF by polymerase chain reaction (PCR) on day 5. Results of blood examination were within normal ranges, including white blood cells (7180/μl), leukocytes (5220/μl), lymphocytes (1507/μl), monocytes (287/μl), eosinophils (43/μl), and basophils (28/μl). Results of VZV anticomplement immunofluorescence studies revealed values of 19 mg/dl for immunoglobulin G (IgG) and below 1 mg/dl for IgM, which indicates the previous infection and acquisition of humoral immunity against VZV. Moreover, there was no increase in the inflammatory biomarker levels. She had normal levels of quantitative immunoglobulins and lymphocyte markers: IgG 1106 mg/dl (normal range, 870–1700 mg/dl), IgA 71 mg/dl (normal range, 110–410 mg/dl), IgM 132 mg/dl (normal range, 46–260 mg/dl), CD3 71.6% (normal range, 59–88%), CD4 32.1% (normal range, 29–65%), CD8 38.8% (normal range, 13–40%), CD4/CD8 ratio 0.83 (normal range, 0.9–3.2), CD19 12.8% (normal range, 4–26%), and CD56 15.5% (normal range, 2–26%).

She was diagnosed with aseptic meningitis and cutaneous manifestation of herpes zoster despite immunocompetence. Acyclovir (45 mg/kg/day) was administered intravenously for 3 days from admission. Because the symptoms of headache, neck stiffness, and skin rash eventually resolved, treatment was switched to oral valacyclovir (75 mg/kg/day) for another 10 days. Given the favorable evolution of the illness, she was discharged from the hospital on day 8. She had no apparent sequelae or comorbidities at the time of the 6-week follow-up.

## Discussion and conclusions

Herpes zoster is caused by the reactivation of VZV that remains in the sensory ganglion cells after primary infection of varicella or vaccination. Waning of cell-mediated immunity to VZV declines in the elderly or in the immunosuppressive state; this is thought to trigger the reactivation of herpes zoster because antibody remains unchanged or even increases with age [1, 2]. Therefore, the incidence of herpes zoster infection is generally considered rare in immunocompetent people and in children.

The distribution of the age at onset of herpes zoster among immunocompetent and immunocompromised children was similar. Both had two peaks, namely at 4–5 years and 10–13 years of age [3]. Another report showed a different tendency with a single peak at 10–14 years of age [4].

Development of neurological complications such as meningitis in varicella zoster is believed to be rare. In a study of 859 adult patients with herpes zoster, meningitis was reported in only 0.5% cases [1]. Authors of another report reviewed 92 cases of children with herpes zoster and found 5 patients with meningitis (frequency, 5.4%) [2]. Therefore, herpes zoster with aseptic meningitis in immunocompetent children seems to be rare. Similar cases have been published earlier, all of which were related to a history of varicella and reactivation of wild-type VZV [5–8] (Table 1).

**Table 1:** Reported cases of herpes zoster and meningitis in immunocompetent children with wild-type varicella zoster virus

Case No.	Age, sex	Onset of varicella	Clinical presentation	Treatment and outcome	Reference
1	3 years, F	2 months	Trigeminal skin rash and meningitis		[5]
2	13 years, F	2 years	Trigeminal skin rash and meningitis		[5]
3	15 years, M	5 years	Thoracic skin rash and meningitis		[5]
4	12 years, M	19 months	Only severe headache	Any antiviral drug was given, and patient recovered	[6]
5	8 years, M	6 months	Painful skin rash in right thoracic area and headache	Treated with acyclovir for 7 days, and patient recovered	[7]
6	14 years, M	3 years	Maculopapular rash on left dorsal skin, mild fever, and headache	Treated with acyclovir 30 mg/kg/day for 10 days, and patient recovered	[8]
7	11 years, F	2 years	Skin rash and headache	Treated with acyclovir 45 mg/kg/day for 4 days, oral valacyclovir 75 mg/kg/day for another 10 days, and patient recovered	Our patient

*F* female, *M* male

For diagnosis, PCR analysis of viral particles in the CSF is crucial when aseptic meningitis is suspected. A rise in antibody titer between paired serum samples might also be helpful. Although dermatomal distribution of vesicular skin lesions is a typical symptom of varicella, an atypical case without any skin lesions has also been reported [6]. Thus, VZV should be raised as a pathogen of meningitis even without typical skin rash. Notably, recent reports have also described diagnosis of meningitis in the reactivation of vaccine strain VZV in immunocompetent children [9, 10] (Table 2). Both wild-type and vaccine strain VZV may be recognized as causes of meningitis in children. Generally, herpes simplex virus, enterovirus, cytomegalovirus, and Epstein-Barr virus should be listed as pathogens of meningitis in children; however, in our patient, we conducted a PCR examination for VZV only.

**Table 2:** Reported cases of herpes zoster and meningitis in immunocompetent children with varicella zoster virus vaccine strain

Case No.	Age	Clinical presentation	Treatment and outcome	Reference
1	4 years old	Skin rash on right arm and meningitis	Treated with acyclovir and patient recovered	[9]
2	8 years old	Skin rash on left shoulder and meningitis	Treated with acyclovir 45 mg/kg/day for 7 days and patient recovered	[9]
3	9 years old	Skin rash at left C5–C6 dermatome and meningitis	Treated with acyclovir (1500 mg/m^2^/day) for 8 days and patient recovered	[9]
4	12 years old	Skin rash at left C5–C6 dermatome and meningitis	Treated with acyclovir for 7 days and patient recovered	[9]
5	7 years old	Right arm rash and pain and fever, headache, photophobia, and vomiting	Treated with acyclovir for 21 days and patient recovered	[10]

The optimal therapy for meningitis with varicella zoster infection has not been determined yet. However, the guidelines issued by the Infectious Diseases Society of America recommend the administration of intravenous acyclovir at 10–15 mg/kg every 8 h for VZV encephalitis [11]. In our patient, the antiviral therapy was given for 14 days. The clinical prognosis of VZV meningitis seems to be appreciable, considering our patient’s case and other case reports [5–10]. No neurological sequelae have been reported.

No specific immune deficiency was identified in our patient from either her medical history or blood examinations, such as immunoglobulins and several lymphocyte markers. Currently, we have no data to explain the co-occurrence of VZV reactivation and meningitis in the immunocompetent child. Several reports have suggested that VZV infection in the first year of life could be a risk factor for herpes zoster [5, 12]. Low specific immune response, especially cellular response, due to the immature immune system in the first year of life is assumed to be the main reason for VZV reactivation in an immunocompetent child [12]. However, our patient had a history of chickenpox at the age of 2 years. Moreover, only two of seven patients reported had been diagnosed with chickenpox during the first year of life (Table 1). As another trigger of herpes zoster reactivation, our patient’s feeling of fatigue due to exhaustive preparation for school gymnastic events over several weeks should be considered. Although we could not find a similar case report emphasizing fatigue as a key trigger of herpes zoster infection, there might be a relationship between these parameters in immunocompetent children. Interestingly, the state of puberty might be another trigger for herpes zoster infection. The level of estrogen, which is known as an inhibitor of cell-mediated immunity, rises rapidly at the beginning of puberty. Also, it is reported that the refusal response to allogenic skin graft is inhibited in mouse with pituitary excision [13]. However, we did not examine the role of pituitary hormones, such as estrogen, gonadotropin, and adrenocorticotropic hormone, which may reflect the state of puberty. Nevertheless, it is necessary to examine not only several lymphocyte markers but also the aforementioned hormones in order to clarify the factors affecting rare immune deficiencies in immunocompetent children.

In conclusion, neurological complications such as meningitis due to VZV reactivation are uncommon, especially in an immunocompetent child. No specific immune deficiency was identified in our patient. It may not be too difficult for an expert to list VZV as a cause of meningitis in immunocompetent children; however, it might be tough for a young trainee because aseptic meningitis with VZV is rare. We conclude that, although rare, VZV should be recognized as a potential cause of viral meningitis in immunocompetent children.

## Abbreviations

CSF: Cerebrospinal fluid
IgG: Immunoglobulin G
IgM: Immunoglobulin M
PCR: Polymerase chain reaction
VZV: Varicella zoster virus
